# Potential Sources and Transmission of *Salmonella* and Antimicrobial Resistance in Kampala, Uganda

**DOI:** 10.1371/journal.pone.0152130

**Published:** 2016-03-21

**Authors:** Josephine A. Afema, Denis K. Byarugaba, Devendra H. Shah, Esther Atukwase, Maria Nambi, William M. Sischo

**Affiliations:** 1 Department of Veterinary Clinical Sciences, College of Veterinary Medicine, Washington State University, Pullman, WA, United States of America; 2 Department of Biomolecular Resources and Biolaboratory Sciences, College of Veterinary Medicine and Animal Resources and Biosecurity, Makerere University, Kampala, Uganda; 3 Department of Veterinary Microbiology and Pathology, College of Veterinary Medicine, Washington State University, Pullman, WA, United States of America; Ross University School of Veterinary Medicine, SAINT KITTS AND NEVIS

## Abstract

In sub‒Saharan Africa, non‒typhoidal *Salmonellae* (NTS) cause invasive disease particularly in children and HIV infected adults, but the disease epidemiology is poorly understood. Between 2012 and 2013, we investigated NTS sources and transmission in Kampala. We detected *Salmonella* in 60% of the influent and 60% of the effluent samples from a wastewater treatment plant and 53.3% of the influent and 10% of the effluent samples from waste stabilization ponds that serve the human population; 40.9% of flush‒water samples from ruminant slaughterhouses, 6.6% of the poultry fecal samples from live bird markets and 4% of the fecal samples from swine at slaughter; and in 54.2% of the water samples from a channel that drains storm–water and effluents from the city. We obtained 775 *Salmonella* isolates, identified 32 serovars, and determined resistance to 15 antimicrobials. We genotyped common serovars using multiple‒locus variable number tandem repeats analysis or pulsed‒field gel electrophoresis. In addition, we analyzed 49 archived NTS isolates from asymptomatic livestock and human clinical cases. *Salmonella* from ruminant and swine sources were mostly pan‒susceptible (95%) while poultry isolates were generally more resistant. *Salmonella* Kentucky isolated from poultry exhibited extensive drug resistance characterized by resistance to 10 antimicrobials. Interestingly, similar genotypes of *S*. Kentucky but with less antimicrobial resistance (AMR) were found in poultry, human and environmental sources. The observed AMR patterns could be attributed to host or management factors associated with production. Alternatively, *S*. Kentucky may be prone to acquiring AMR. The factors driving AMR remain poorly understood and should be elucidated. Overall, shared genotypes and AMR phenotypes were found in NTS from human, livestock and environmental sources, suggesting zoonotic and environmental transmissions most likely occur. Information from this study could be used to control NTS transmission.

## Introduction

Non–typhoidal *Salmonellae* (NTS) are estimated to cause 93.8 million cases of gastroenteritis [1] and 3.4 million cases of invasive disease [2] in humans every year, thereby exerting a huge burden on global public health. In developed countries, food animals constitute an important reservoir, and most human illnesses are foodborne [1]. Some human illnesses are attributed to contact with food animals and pets [3,4], and rare incidences of waterborne and environmental transmission occur [3]. However, in sub‒Saharan Africa, the epidemiology of NTS is still poorly understood [5]. One study has shown evidence for asymptomatic human carriers of NTS and human–to–human transmission [6]. Another study has shown that *Salmonella* Typhimurium ST313 is undergoing microevolution to adapt to the human population in Africa, implying humans may be the source of infection for this strain [7]. The role of animals in the epidemiology of NTS is not yet clearly defined. Some studies suggest animals or the environment (soil, water and animals) may not be significant reservoirs of NTS for humans [6,8]. However, detection of *Salmonella* Typhimurium DT56 with similar PFGE and antimicrobial resistance (AMR) patterns in poultry and humans suggests zoonotic infections occur in sub–Saharan Africa [9]. Furthermore, finding *Salmonella* serovars Albany, Hadar, Heidelberg, and Virchow with indistinguishable PFGE patterns, but *S*. Enteritidis, *Salmonella* Infantis and *S*. Typhimurium with distinct PFGE patterns in poultry and humans suggests poultry are the source of some, but not the predominant serovars associated with human salmonellosis [10]. Nosocomial infections have also been reported [11]. Inadequate access to clean water and sanitation in developing countries implies waterborne transmission of NTS is likely greater than foodborne transmission [1]. In Uganda, several communities lack access to clean water, effective sanitation and proper waste management [12] raising the possibility of NTS transmission via contaminated water and environments. *Salmonella* can persist in the environment for several months to years [13]; hence, the environment may play a key role in transmission.

Invasive non–typhoidal salmonellosis is considered an emerging and neglected tropical disease partly due to limited knowledge on disease epidemiology [14], yet a good understanding of transmission pathways is crucial for disease control and prevention [5]. This study was therefore designed to investigate potential sources of NTS in Kampala, Uganda by determining occurrence in human, livestock and environmental sources. In addition, we analyzed AMR and genotypic structure in common serovars in order to infer transmission. Additional information on transmission was gained by comparative analysis of NTS collected in this study and archived human and livestock isolates from a previous study.

## Materials and Methods

### Ethic statement

No human subjects were involved and no vertebrate animals were involved. Research permits were granted by Uganda National Council for Science and Technology, permit number NS414.

### Study area and sampling

**Fig 1:**
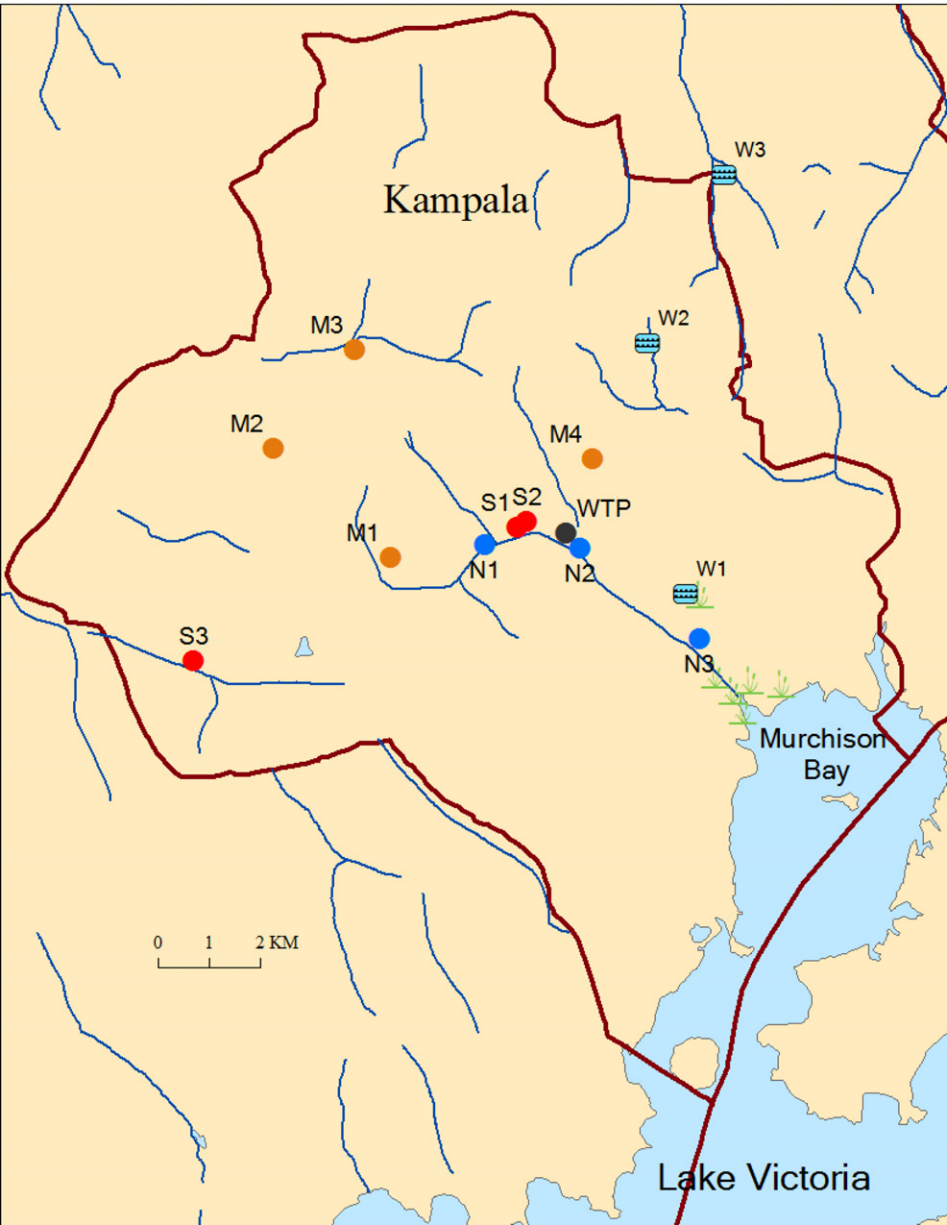
Map of Kampala showing the 14 sampling sites. M1–M4: live poultry markets; N1–N3: sampling sites along Nakivubo Channel; S1–S2: ruminant slaughterhouses; S3: swine slaughterhouse; WTP: wastewater treatment plant; W1–W3: waste stabilization pond systems.

This study was conducted in Kampala, the capital and largest city in Uganda. Samples were collected at 14 sites representing human, livestock and environmental sources (Fig 1). At the beginning of the study (July‒August 2012), samples were collected from each site every 7‒14 days, but from September 2012‒February 2013, samples were collected once a month.

#### Human source samples

Kampala has a population of 1,516,210 people [15]. Human wastewater from this community was collected from a wastewater treatment plant (WTP) that serves the city center and vicinities, and from three waste stabilization pond systems (WSP) that serve suburbs. The WTP is located at Bugolobi (N00.31929 E032.60633), and the WSP designated W1, W2 and W3 are located at Bugolobi (N00.30645 E032.62903), Ntinda (N00.35062 E032.62222), and Naalya (N00.38014 E032.63575) respectively. At the WTP, we collected influent (1L) and secondary effluent (1L) samples at the point of inflow and discharge respectively. Waste stabilization pond systems W1, W2 and W3 consist of two, three and four connected ponds respectively [16]. At each WSP, influent (1L) and effluent (1L) samples were collected from the first and last ponds respectively.

#### Livestock samples

We collected livestock samples from two ruminant slaughterhouses (S1 and S2) located at N00.31811 E032.59933 and N00.31909 E032.60094, a swine slaughterhouse (S3) located at Wambizi (N00.29467 E32.54264), and four live poultry markets (M1, M2, M3 and M4) located at Nakasero (N00.31283 E032.57722), Kasubi (N00.33194 E032.55672), Kalerwe (N00.34928 E032.57092) and Nakawa (N00.33014 E032.61271) respectively. The ruminant slaughterhouses process approximately 250 head of cattle and 150 goats and sheep daily, and at each facility, we collected flush‒water (1L) at the drainages into sterile plastic bottles. The swine slaughterhouse (S3) processed approximately 70‒150 pigs daily. At S3, we collected fecal droppings on holding cage floors using cotton tipped swabs, and pooled two swabs from different droppings into 10ml buffered peptone water (BPW). In addition, we swabbed the slaughter floor and pooled 2 swabs into 10ml BPW. Similarly, at the live poultry markets, we collected fresh feces from cage floors.

#### Environmental samples

Environmental samples were collected at three points along Nakivubo Channel (NC) designated N1 (N00.31505 E032.59370), N2 (N00.31449 E032.61039) and N3 (N00.29854 E032.63136). Nakivubo channel drains storm–water from the city, as well as wastewater from various sources including the WTP and slaughter facilities, and it empties into Murchison Bay in Lake Victoria (Fig 1). Site N1 is upstream of the WTP and ruminant abattoirs, N2 is midstream, and N3 is downstream. At each site, we collected water samples (1L) into sterile plastic bottles.

### *Salmonella* isolation and serotyping

All samples were kept in a cool‒box with cold packs and processed within 24 hours of collection at the Microbiology Laboratory, College of Veterinary Medicine, Makerere University. Liquid samples were processed using previously described methods [17] with minor modifications. Briefly, duplicate sample sets of 3 ml and 40 ml were transferred into 50 ml conical tubes and centrifuged at 10,000 x g for 10 min. The supernatant was removed and 40 ml of tetrathionate broth (TTB, Hardy Diagnostics, USA) was added to the first sample set and incubated at 37°C for 16‒24 hours. To the second sample set, 40 ml of Rappaport‒Vasilliadis (R10, Hardy Diagnostics, USA) broth was added and incubated at 42°C for 16‒24 hours. Enriched broths were streaked onto Xylose‒Lysine‒Deoxycholate (XLD, Hardy Diagnostics, USA) or Xylose‒Lysine‒Tergitol‒4 (XLT‒4, Difco Laboratories) selective agar plates and incubated at 37°C for 16‒24 hours. Enriched broths suspected to contain *Salmonella* (based on suspected colonies on XLD or XLT–4) were subjected to serial dilutions [18] and re‒plated onto XLD or XLT‒4 agar plates to obtain well isolated colonies. Up to 5 *Salmonella* suspected colonies were picked from each plate and stored on trypticase soy agar (Hardy Diagnostics, USA) or brain heart infusion agar (Hardy Diagnostics, USA) at room temperature.

The samples in 10 ml BPW were incubated at 37°C for 16‒24 hours, then 1 ml of BPW was added to 9 ml of R10 enrichment broth and incubated at 42°C for 16‒24 hours. Enriched R10 broths were streaked onto XLD or XLT‒4 agar plates and processed as above. All isolates were shipped to Washington State University (WSU) for further processing in compliance with Uganda National Council of Science and Technology regulations and materials transfer agreement with Makerere University.

Suspect *Salmonella* isolates were confirmed by PCR amplification of the *inv*A gene [19]. All isolates were subsequently serotyped using slide agglutination with antisera and serovar determined by the White-Kauffmann-Le Minor scheme[20].

### Antimicrobial resistance testing

Antimicrobial resistance testing was performed on isolates obtained from human and livestock sources using the agar dilution assay, and on environmental-source isolates using the disc diffusion assay. Fifteen antimicrobials were used: amikacin, amoxicillin–clavulanic acid, ampicillin, cefotaxime, cefoxitin, ceftiofur, chloramphenicol, ciprofloxacin, gentamicin, kanamycin, nalidixic acid, streptomycin, sulfisoxazole, tetracycline, and trimethoprim–sulfamethoxazole [21,22].About 6% of the isolates that were tested using the agar diffusion assay were retested with the disc diffusion technique. Quality control organisms from the American Type Culture Collection (*E*. *coli* ATCC 25922, *Pseudomonas aeruginosa* ATCC 27853, *Salmonella* Typhimurium ATCC 29945 and *Staphylococcus aureus* ATCC 25923); and from the *Salmonella* Bank at Washington State University (*Salmonella* Newport S13990 and *Salmonella* Typhimurium S8740) were included in each batch. Isolates were categorized as susceptible or resistant to each antimicrobial based on the Clinical Laboratory Standards Institute (CLSI) definitions for *Enterobacteriaceae* or *Salmonella* [22]except for streptomycin where the National Antimicrobial Resistance Monitoring System (NARMS) breakpoint was used [23].

#### Comparison of single AMR

To compare AMR from different sources, we used a Bayesian method with a minimally informative beta prior, [U(0,1) = (beta(1, 1))], and posterior distributions were estimated with a beta‒binomial distribution as (# resistant isolates + 1, # susceptible isolates + 1) [24].

#### Latent class analysis of AMR

Latent class analysis (LCA) was used to determine AMR structure or patterns in the overall resistance results for the isolates. LCA is a statistical method that is used to reduce large and complex categorical datasets into fewer categories that reveal important patterns within data [25]. LCA has been used to characterize AMR in *Escherichia coli* from dairy cattle [26]. The model notation for LCA is well documented [25,27]. Two parameters are estimated in a latent class model; gamma (γ), the probability of latent class membership, and rho (ρ), the probabilities of item response conditional on class membership. Item response probability is the “probability of a particular observed response on a particular variable conditional on class membership” and it is used to interpret latent classes [25].

In this study, latent class membership is the proportion of isolates in each latent class, and item response probability is the probability an isolate in a given latent class is resistant to an antimicrobial. The basic LCA can be extended to include multiple groups to test differences in latent class prevalence and item response probabilities across groups. We used LCA with multiple groups (human, livestock, and the environment) to test differences in AMR structure across groups. The analysis was done using SAS PROC LCA [27], and model selection was based on tests of absolute model fit, assessment of relative model fit, parsimony and ease of interpretability [25].

### Genotyping

#### Multiple‒Locus Variable number tandem repeats Analysis (MLVA)

*Salmonella* Typhimurium and *S*. Enteritidis were genotyped using MLVA [28–31] according to PulseNet protocols. Briefly, DNA was prepared as boiled cell lysate and seven variable number tandem repeats (VNTR) loci were amplified in two multiplex PCR reactions. The forward primers were fluorescently labeled at the 5’ end to aid separation. In *S*. Typhimurium, four loci (ST3, ST5, ST7 and STTR10pl) were amplified in one reaction and three loci (ST2, ST6 and ST8) in another reaction. In *S*. Enteritidis, four loci (SE1, SE2, SE8 and SE6), and three loci (SE5, SE3 and SE9) were similarly amplified.

The labeled PCR amplicons were prepared for fragment analysis and separated by capillary electrophoresis using Applied Biosystems Genetic Analyzer 3730xl at the WSU Laboratory for Biotechnology and Bioanalysis. The copy number (allele) for each VNTR locus was determined as follows: (observed fragment size—offset)/repeat size. Based on alleles at the 7 loci, isolates were assigned a genotype number and MLVA data was analyzed using PHYLOViZ software [32]. We used goeBURST algorithm and its minimum spanning tree to visualize evolutionary relationships between genotypes and to infer transmission.

#### Pulsed field gel electrophoresis (PFGE)

With the exception of *S*. Typhimurium and *S*. Enteritidis, PFGE was used to subtype isolates of serovars recovered from more than one source. We adapted the PulseNet protocol [33] and cleaved DNA using restriction endonuclease *XbaI* (Fermentas, Thermo Fisher Scientific). The PFGE bands were assigned and analyzed using BioNumerics 6.6 software (Applied Maths, Austin, TX, USA). Isolates with ≥85% band pattern similarity based on the Dice coefficient similarity index were considered closely related. Cluster analysis was performed by the unweighted pair group method with arithmetic mean, band matching tolerance of 2%, and relaxed doublet matching.

## Results

### *Salmonella* detection and serovar distribution

Overall, *Salmonella* was commonly detected (Table 1) in human influent from the WTP (60%) and WSP (mean = 53.3%), NC water (mean = 54.2%), and flush‒water from the ruminant slaughterhouses (mean = 40.9%). *Salmonella* was also detected in effluent from the WTP (60%) and W1 (30%), swine slaughter floor (11.5%), and fecal samples from swine (4%) and poultry (6.6%). A total of 775 *Salmonella* isolates were obtained from all sources and retained for further analyses (S1 File). The median (and range) of the number of *Salmonella* colonies obtained per sample were as follows: WTP, 12 (1–29); WSP, 9.5 (4–20); NC, 10.5 (3–16); ruminant slaughterhouses, 8 (1–17); swine fecal sample, 2 (1–8); and poultry fecal sample, 6 (1–12).

**Table 1:** *Salmonella* detection from various sources in Kampala, Uganda.

Sample & sampling site	# Samples	# Positive	% Positive
Wastewater treatment plant			
Influent	10	6	60.0
Effluent	10	6	60.0
Influent from waste stabilization ponds			
W1	10	6	60.0
W2	10	3	30.0
W3	10	7	70.0
Total	30	16	53.3
Effluent from waste stabilization ponds			
W1	10	3	30.0
W2	10	0	0.0
W3	10	0	0.0
Total	30	3	10.0
Water from Nakivubo Channel			
N1	9	4	44.4
N2	6	4	66.7
N3	9	5	55.6
Total	24	13	54.2
Flush–water from ruminant slaughterhouses			
S1	11	5	45.5
S2	11	4	36.4
Total	22	9	40.9
Swine slaughterhouse (S3)			
Fecal samples	99	4	4.0
Slaughter floor swabs	26	3	11.5
Fecal samples from live poultry markets			
M1	99	2	2.0
M2	83	6	7.2
M3	100	11	11.0
M4	99	6	6.1
Total	381	25	6.6

We identified a total of 32 *Salmonella* serovars from all sources (Table 2). The common serovars included *Salmonella* Enteritidis, *Salmonella* Haifa, *Salmonella* Heidelberg, *Salmonella* II 42: r:-, *Salmonella* Kentucky, *Salmonella* Newport, *Salmonella* Senftenberg, *Salmonella* Stanleyville, *Salmonella* Typhimurium and *Salmonella* Virchow. Interestingly, we found few *S*. Typhimurium isolates in human wastewater and did not detect *S*. Enteritidis.

**Table 2:** *Salmonella* serovars detected from various sources in Kampala, Uganda.

Serovar	NC	Human	Ruminants^a^	Swine	Poultry	Total
Kentucky	20	44	0	0	84	148
Stanleyville	45	51	4	0	0	100
Newport	19	8	34	12	0	73
Haifa	3	62	0	0	0	65
Heidelberg	4	44	0	0	8	56
II 42:r:-	3	31	18	0	4	56
Enteritidis	14	0	1	0	9	24
Senftenberg	12	12	0	0	0	24
Typhimurium	7	2	0	0	14	23
Virchow	6	1	0	0	16	23
Os	0	21	0	0	0	21
Aberdeen	1	19	0	0	0	20
Agona	0	20	0	0	0	20
Poona	0	15	0	0	0	15
9,12:a:2	0	4	6	0	0	10
Zanzibar	10	0	0	0	0	10
Abony	0	0	9	0	0	9
Chandans	9	0	0	0	0	9
Fulica	0	0	0	8	0	8
Guildford	0	0	0	8	0	8
Mbandaka	7	0	0	0	0	7
Plymouth	0	7	0	0	0	7
Kallo	0	0	0	0	6	6
Litchfield	0	5	0	1	0	6
Untypeable	5	0	0	0	1	6
Havana	0	4	0	0	0	4
28:z35:-	0	3	0	0	0	3
Coleypark	0	0	0	4	0	3
Damman	0	1	0	2	0	3
Kitenge	0	0	0	3	0	3
Unknown	0	1	0	0	0	1
Muenchen	0	1	0	0	0	1
Paratyphi B	1	0	0	0	0	1
Saintpaul	0	0	0	0	1	1
Total	166	356	72	38	143	775

NC, Nakivubo Channel

^a^ Ruminants includes cattle, goats and sheep

### Antimicrobial resistance

#### Resistance to single drugs

Overall, 475 out of 775 isolates (61.3%) were susceptible to all 15 antimicrobials (pan‒susceptible). There was no resistance to amikacin, cefotaxime and ceftiofur, and only one isolate was resistant to cefoxitin and gentamicin. However, resistance to nalidixic acid (31.1%), sulfisoxazole (31%), tetracycline (23.8%), ciprofloxacin (17.8%), trimethoprim/sulfamethoxazole (16.5%) and streptomycin (15%) was common (S1 Table).

**Fig 2:**
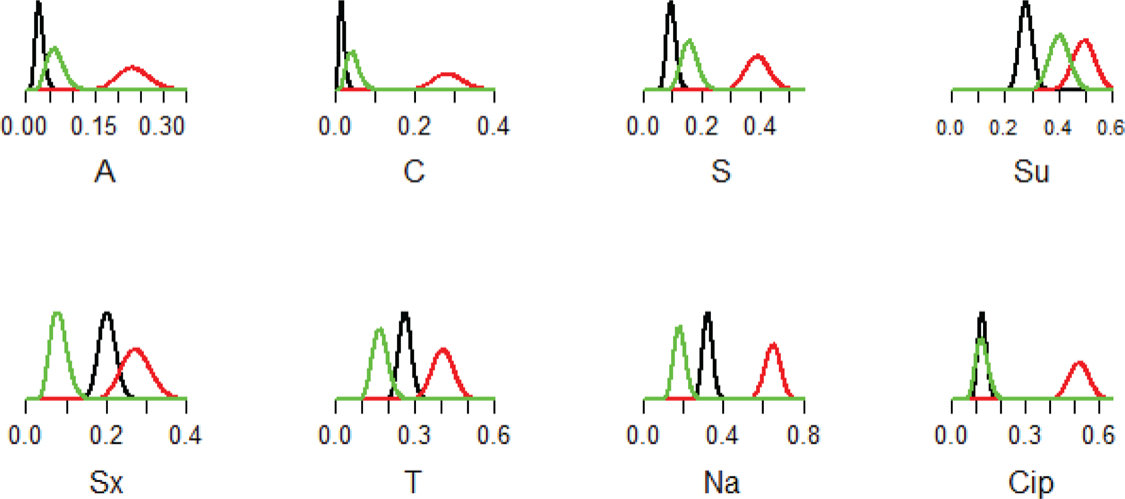
Plots of the posterior distributions of the proportion of *Salmonella* from human wastewater (black), poultry (red) and Nakivubo Channel (green) that were resistant to eight antimicrobials: A, ampicillin; C, chloramphenicol; S, streptomycin; Su, sulfisoxazole; Sx, trimethoprim/sulfamethoxazole; T, tetracycline; Na, nalidixic acid and Cip, ciprofloxacin.

When AMR was evaluated according to source, ruminant and swine isolates were mostly pan‒susceptible, whereas resistance to ampicillin, chloramphenicol, quinolones, streptomycin, sulfonamides and tetracycline was detected in environmental, poultry and human source isolates (Fig 2). Poultry isolates had additional resistance to amoxicillin/clavulanic acid and kanamycin, and were generally more resistant compared to other source isolates.

Across all sources, *S*. Kentucky had diverse resistance profiles and tended to exhibit extensive drug resistance. For instance, the SSuTCipNa resistance profile was shared by *S*. Kentucky from human, environmental and poultry sources, however, a deca-resistant profile, ACKSSuSxTAmcCipNa was unique to poultry (S2 Table). While *S*. Kentucky isolated from poultry were multi-drug resistant, other serovars found in poultry were pan‒ susceptible or had low level resistance (S3 Table).

#### Latent class analysis of AMR structure

Additional information on AMR structure across source was obtained using LCA. We attempted LCA with multiple groups (NC, human and poultry), but we observed differences in interpretation of some latent classes. We therefore performed separate analysis for each group and selected the best fitting model (S4 Table) using the G^2^ statistic, the Akaike Information Criterion, or the Bayesian Information Criterion [25]. We did not perform LCA for ruminant and swine isolates because they were mostly pan–susceptible.

Our models showed that AMR in *Salmonella* from environmental, human, and poultry sources was best described by 3, 4 and 5 latent classes respectively (Table 3). The class labels indicate resistance to antimicrobials in that class, for instance the “SSuT+ quinolones” class has high probabilities of resistance to streptomycin, sufisoxazole, tetracycline, and quinolones (ciprofloxacin and nalidixic acid). The “pan‒susceptible” and “SSuT+ quinolones” classes were common to all sources, while the “quinolones” and “SuSxTNa” (sufisoxazole, trimethoprim-sulfamethoxazole, tetracycline and nalidixic acid) or “SSuSxTNa” (additional resistance to streptomycin) classes occurred in human and poultry source isolates. The “ACSSuSx” (ampicillin, chloramphenicol, streptomycin, sufisoxazole, and trimethoprim-sulfamethoxazole) class was exclusive to environment source isolates while the “deca‒resistant” class characterized by resistance to 10 antimicrobials was exclusive to poultry isolates.

**Table 3:** Antimicrobial resistance structure in *Salmonella* from various sources revealed by Latent class analysis.

	Nakivubo Channel (n = 165)			Human source (n = 356)				Poultry (n = 143)				
Latent class	PS	SSuT+	ACSSuSx	PS	SuSxTNa	SSuT+	Quinolones	PS	SSuT+	Deca-	Quinolones	SSuSxTNa
		quinolones				quinolones			quinolones	resistant		
Prevalence	79.8%	14.9%	5.3%	65.9%	18.6%	9.6%	5.9%	36.9%	22.4%	19.3%	13.2%	8.4%
Antimicrobials												
A	0.015	0.001	0.900^a^	0.000	0.076	0.146	0.000	0.001	0.095	0.995^a^	0.003	0.183
Amc	0.000	0.000	0.000	0.000	0.000	0.029	0.000	0.001	0.001	0.994^a^	0.002	0.004
C	0.000	0.041	0.675^a^	0.000	0.000	0.146	0.048	0.001	0.374	0.995^a^	0.003	0.005
K	0.000	0.000	0.000	0.000	0.000	0.029	0.000	0.001	0.002	0.995^a^	0.002	0.540
S	0.000	0.807^a^	0.680^a^	0.000	0.000	0.993^a^	0.001	0.002	0.655^a^	0.996^a^	0.004	0.632^a^
Su	0.254	0.991^a^	0.978^a^	0.000	0.997^a^	0.966^a^	0.003	0.021	0.997^a^	0.997^a^	0.005	0.902^a^
Sx	0.000	0.179	0.965^a^	0.000	0.997^a^	0.148	0.049	0.001	0.002	0.995^a^	0.003	0.987^a^
T	0.015	0.946^a^	0.295	0.000	0.967^a^	0.878^a^	0.003	0.002	0.593^a^	0.996^a^	0.004	0.989^a^
Cip	0.008	0.768^a^	0.005	0.000	0.001	0.848^a^	0.713^a^	0.002	0.997^a^	0.961^a^	0.786^a^	0.009
Na	0.060	0.891^a^	0.007	0.001	0.982^a^	0.849^a^	0.989^a^	0.097	0.998^a^	0.998^a^	0.995^a^	0.815^a^

^a^ An isolate in a given latent class has a ≥0.5 probability of being resistant to a given antimicrobial and this information is used to assign class labels.

A, ampicillin; Amc, amoxicillin/clavulanic acid; C, chloramphenicol; K, kanamycin; S, streptomycin; Su, sufisoxazole; Sx, trimethoprim/sulfamethoxazole; T, tetracycline; Cip, ciprofloxacin;Na, nalidixic acid and PS, pan-susceptible.

### Archived *Salmonella* isolates from humans and livestock

We included 49 archived isolates from the College of Veterinary Medicine, Makerere University (S2 File) for comparative analysis. These isolates came from samples obtained from apparently healthy poultry at a commercial poultry farm and a market, cattle and caprine in slaughterhouses, and human clinical cases in two hospitals in Kampala. We selected archived isolates that belonged to serovars (Table 4) shared with isolates we collected (Table 2).

**Table 4:** Archived *Salmonella* isolates collected from Uganda between 2003 and 2010.

Serovar	Poultry	Cattle	Caprine	Human	Total
Enteritidis	2	0	0	9	11
Haifa	18	0	1	1	20
Stanleyville	0	1	2	7	10
Typhimurium	2	0	0	6	8
Total	22	1	3	23	49

### Genotyping

#### MLVA and AMR in S. Enteritidis

**Fig 3:**
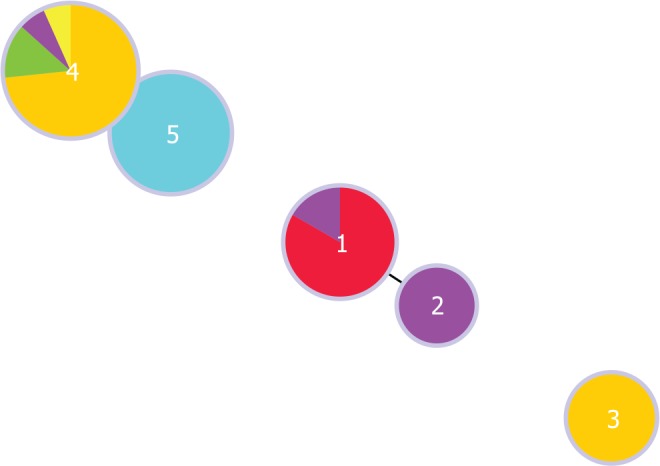
Clusters of MLVA genotypes produced by goeBURST algorithm at the single locus variant level for 34 *Salmonella* Enteritidis isolates. Each genotype is represented by a circle and the size of the circle is proportional to the number of isolates on a log scale. The source of the isolates are color coded: archived human clinical isolate from blood (red) or feces (purple); environment (orange); cattle (yellow), poultry farm (green) and poultry market (light blue).

We performed MLVA on 23 *S*. Enteritidis isolates from this study and 11 archived isolates, and we identified 5 genotypes. To establish genotypic relationships, we performed cluster analysis at the single locus variant level (Fig 3). Genotypes 1 and 2 clustered together and this cluster comprised archived human clinical isolates with similar resistance profiles (ACSSuSxT and ACSSuSxTNa). Genotype 3 remained separate and it comprised environmental isolates with Su resistance profile. Genotype 4 was found in multiple sources (NC = 11, and archived cattle = 1, poultry = 2, and human = 1), while genotype 5 occurred in poultry (n = 5). Genotypes 4 and 5 formed another cluster and the isolates were pan–susceptible.

#### MLVA and AMR in S. Typhimurium

**Fig 4:**
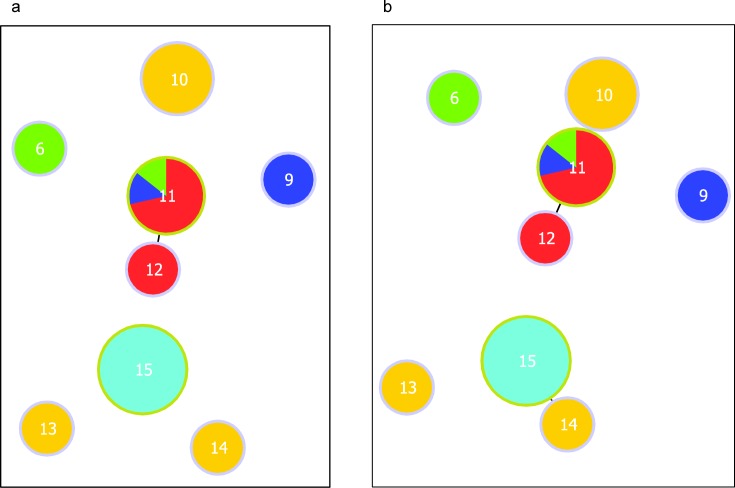
**Clusters of MLVA genotypes belonging to 31 *Salmonella* Typhimurium isolates at the a) single locus variant, and b) double locus variant level.** Each genotype is represented by a circle, the number inside the circle is the genotype name, and the size of the circle is proportional to the number of isolates on a log scale. The source of the isolates is color coded: human clinical (red); human influent (dark blue); NC (orange); archived poultry (green) and poultry from this study (light blue).

We performed MLVA on 23 *S*. Typhimurium isolates from this study and 8 archived isolates, and we detected 8 genotypes. Each genotype belonged to isolates from a single source except genotype 11 which was found in diverse sources. *S*. Typhimurium genotype 6 (archived poultry), genotype 9 (human influent), genotype 10 (NC) and genotype 11 (human influent, archived poultry and human clinical) had the same resistance profile (ACSSuSx). Genotype 12 detected in archived human clinical isolate had ACSSxT resistance profile, and genotype 13 detected in NC isolate had SSuT resistance. When clustering was performed at the single locus variant level, genotypes 11 and 12 clustered together while other genotypes remained separate (Fig 4A). At the double locus variant level, genotypes 10, 11, and 12 formed a cluster comprising multidrug resistant isolates, while genotypes 14 (NC) and 15 (poultry) formed a cluster comprising pan‒susceptible isolates (Fig 4B).

#### PFGE typing

We performed PFGE on isolates of 8 serovars that were recovered from more than one source (Table 5). We found *Salmonella* Haifa with similar PFGE (≥85% band pattern similarity) and resistance (SuSxTNa) patterns across diverse sources. For instance, PFGE pattern designated 1 was detected in various sources, sites and sampling times. It was also concurrently (same day) detected in WTP effluent and N2. Similarly, most *S*. Heidelberg isolates had similar PFGE patterns, were pan‒susceptible, and came from diverse sources and sites (WTP, N1, W1 and M3). Also, most *Salmonella* II 42:r:‒ isolates from various sources had similar PFGE and resistance patterns. Overall, *S*. Newport from various sources had similar PFGE patterns and the isolates were pan–susceptible.

**Table 5:** PFGE patterns in isolates of common *Salmonella* serovars.

Serovar	PFGE patterns	Date collected	Source & sample	# isolates	Collection site	AMR phenotype
Haifa	1^a^	Jun 2003	Archived poultry	1	Poultry farm	SuSxTNa
		2 Aug 2012	Human effluent	7	WTP	SuSxTNa
		18 Oct 2012	Human effluent	1	WTP	SuSxTNa
			Storm/wastewater	2	N2	SuSxTNa
		19 Oct 2012	Human influent	3	W3	SuSxTNa
		8 Nov 2012	Human influent	6	W1 & W3	SuSxTNa
		21 Feb 2013	Human influent & effluent	8	W1	SuSxTNa
		25 Feb 2013	Storm/wastewater	1	N3	SuSxTNa
	2^a^	Jun 2003	Archived poultry	2	Poultry farm	SuSxTNa
		Sep 2007	Archived caprine	1	Abattoir	SuSxTNa
		2010	Archived poultry	1	Poultry farm	SuSxTNa
		Jan 2010	Archived human clinical	1	Human hospital	SuSxTNa
	3^a^	2 Aug 2012	Human effluent	1	WTP	SuSxTNa
		21 Feb 2013	Human influent	1	W1	SuSxTNa
	4	18 Dec 2012	Human influent	1	WTP	SuSxTNa
Heidelberg	5^b^	22 Feb 2013	Poultry feces	2	M3	PS
	6^b^	20 Aug 2012	Human influent	1	WTP	PS
		11 Sep 2012	Human influent	2	W1	PS
		2 Feb 2013	Storm/wastewater	1	N1	PS
	7^b^	17 Jul 2012	Poultry feces	2	M3	SuSxTNa, PS
		1 Feb 2013	Poultry feces	2	M3	PS
		25 Feb 2013	Storm/wastewater	1	N1	PS
	8^b^	26 Jul 2012	Human influent & effluent	4	W1	PS
	9^b^	16 Jul 2012	Human effluent	4	WTP	PS
II 42:r:-	10^c^	Jul 26 2012	Human influent	4	W3	PS
		Sep 2012	Storm/wastewater	1	N3	PS
		Dec 15 2012	Poultry feces	2	M1	PS
		Jan 28 2013	Human influent	10	W1 & W2	PS
		Feb 21 2013	Human influent	1	W3	PS
		Feb 25 2013	Storm/wastewater	3	N1 & N3	PS
	11^c^	Sep 13 2012	Cattle, sheep, goats	1	S1	PS
		Jan 28 2013	Human influent	1	W2	SSuT+quinolones
		Feb 21 2013	Human influent	2	W2	PS
	12^c^	Oct 18 2012	Cattle, sheep, goats	4	S1	PS
		Dec 18 2012	Cattle, sheep, goats	2	S2	PS
	13	Jan 28 2013	Human influent	1	W1	PS
Kentucky	14^d^	Sep 13 2012	Human effluent	3	WTP	Quinolones
		Oct 18 2012	Storm/wastewater	4	N1	Quinolones, SSuT+ quinolones
				1	N2	SSuT+ quinolones
				4	N3	SSuT+ quinolones
		Dec 15 2012	Poultry feces	3	M2	PS, SSuT+quinolones
		Dec 18 2012	Human influent	2	W1	SSuT+ quinolones
		Jan 28 2013	Human influent & effluent	5	W1	Quinolones, SSuT+ quinolones, deca-resistant
			Human influent	3	W3	Quinolones, SSuT+ quinolones
		Feb 1 2013	Poultry feces	4	M1	SSuT+ quinolones, deca-resistant
				6	M2	Quinolones, deca-resistant
				3	M3	Quinolones
				13	M4	Quinolones, SSuT+ quinolones, deca-resistant
		Feb 2 2013	Storm/wastewater	1	N1	Quinolones
	15^d^	Jan 28 2013	Human effluent	1	W1	SSuT+quinolones
		Feb1 2013	Poultry feces	5	M2	Quinolones, SSuT+ quinolones, deca-resistant
	16^d^	Jan 28 2013	Human influent	2	Influent W2	Quinolones
	17^d^	Feb 1 2013	Poultry feces	4	M2	Quinolones, deca-resistant
	18^d^	Feb2 2013	Storm/wastewater	1	N1	SSuT+ quinolones
	19^d^	Feb 1 2013	Poultry feces	1	M2	Quinolones
	20^d^	Oct 18 2012	Storm/wastewater	1	N2	SSuT+ quinolones
	21^d^	Feb 1 2013	Poultry feces	2	M4	Deca-resistant
	22	Jan 28 2013	Human influent	1	Influent W1	SSuT+ quinolones
Newport	23^e^	9 Aug 2012	Swine abattoir floor swab	5	S3	PS
	24^e^	Jul 23 2012	Cattle, sheep, goats	9	S1 & S2	PS
	25^e^	Jul 18 2012	Storm/wastewater	1	N2	PS
		Aug 20 2012	Cattle, sheep, goats	11	S1 & S2	PS
		Nov 8 2012	Human influent	1	W3	PS
		Dec 15 2012	Human influent	2	W2	PS
		Jan 28 2013	Human influent	1	W3	PS
		Jan 14 2014	Storm/wastewater	1	N3	PS
	26	Jul 18 2012	Storm/wastewater	3	N2	PS
		Feb 2 2013	Storm/wastewater	1	N1	PS
Senftenberg	27	Aug 20 2012	Human effluent	4	WTP	PS
		Feb 2 2013	Storm/wastewater	6	N1	PS
	28	Oct 18 2012	Human influent	2	WTP	PS
		Feb 6 2014	Storm/wastewater	2	N3	PS
	29	Feb 2 2013	Storm/wastewater	1	N1	SuSxTNa
Stanleyville	30^f^	Feb 2009	Archived cattle & caprine	2	Abattoir	PS
		Jan 2010	Archived caprine	1	Abattoir	PS
			Archived human clinical	1	Human hospital	PS
		13 Aug 2012	Human influent & effluent	12	WTP	PS
			Storm/wastewater	7	N2 & N3	ACSSuSx, PS
		20 Aug 2012	Human influent & effluent	9	WTP	PS
		12 Dec 2013	Storm/wastewater	1	N3	ACSuSxT
	31^f^	Aug 13 2012	Storm/wastewater	2	N3	PS
		Aug 20 2012	Human effluent	1	Effluent WTP	PS
		15 Dec 2012	Human influent	1	Influent W2	PS
		12 Dec 2013	Storm/wastewater	4	N3	PS
	32^f^	Jul 2012	Cattle, sheep, goats	3	S1	PS
	33^f^	18 Oct 2012	Human effluent	1	WTP	PS
			Storm/wastewater	1	N2	PS
	34^f^	Apr 2007	Archived human	1	Human hospital	PS
	35^f^	Feb 25 2013	Storm/wastewater	2	N1	PS
	36	13 Sep 2012	Storm/wastewater	3	N3	PS
Virchow	37^g^	Feb 1 2013	Poultry feces	2	M3	PS
	38^g^	Dec 15 2012	Poultry feces	1	M2	PS
	39	Dec 18 2012	Storm/wastewater	3	N3	PS

^a, b, c, d, e, f, g^ PFGE patterns with a Dice similarity index of ≥ 85% in *Salmonella* serovars Haifa, Heidelberg, II 42:r:-, Kentucky, Newport, Stanleyville and Virchow respectively.

Most *S*. Kentucky isolates had similar PFGE patterns but varied resistance phenotypes. While PFGE pattern 14 was widely distributed (all poultry markets, all WSP and N1) and detected during most sampling times, and PFGE pattern 15 was common in poultry and human effluent (W1), the rest of the PFGE patterns occurred at specific sites. Six of the 7 PFGE patterns observed in *S*. Stanleyville were similar and the isolates were mostly pan‒susceptible, apart from few multi–drug resistant (ACSSuSx or ACSuSxT) isolates. The same *S*. Stanleyville PFGE pattern was found in influent and effluent from the WTP and downstream at N2 on the same day, and in archived cattle, caprine and human clinical isolates. Conversely, the PFGE patterns in *S*. Virchow isolates from poultry and the environment were different. The PFGE patterns in *S*. Senftenberg were dissimilar.

## Discussion

We used a community based sampling scheme to determine the occurrence of NTS in various sources, and provide insights into the epidemiology of NTS and associated AMR in Uganda. Unlike studies in the region that concluded animals and the environment (water, soil, sewer, and food) may not constitute an important NTS reservoir for humans [6,8,34], we identified shared serovars, AMR phenotypes, and genotypes (some temporally related) in samples originating from humans, livestock, and the environment. Our results and the detection of *Salmonella* serovars with similar PFGE and antimicrobial resistance patterns in poultry and humans [9,10] suggest zoonotic infections could be important Africa.

We recovered NTS from human wastewater collected from facilities serving communities within Kampala and assumed it originated from the general human population. It is however possible that some of the NTS recovered from human wastewater may have come from nonhuman sources. Our assertion that these samples principally reflect human sources are consistent with a study that recovered *Salmonella* from human influent and concluded wastewater is invaluable for monitoring *Salmonella* and AMR in human populations [17]. Another study detected *S*. Heidelberg in human influent from a small closed community before, during and after an outbreak and recommended ongoing wastewater monitoring to supplement conventional surveillance systems [35].

There was ample evidence for environmental dissemination of *Salmonella* within Kampala. Detection of *Salmonella* at the most upstream site along NC raises the possibility of bacteria flow (in storm‒water) from nonpoint sources such as leaky sewers, septic tanks, improperly constructed pit latrines, humans with no access to toilet facilities, roaming livestock, stray dogs and cats, rodents and wild birds [12]. Concurrent detection of NTS in wastewater originating from the ruminant abattoirs and WTP and downstream in NC suggests these facilities could be important point sources of environmental contamination. Although some plants in Nakivubo wetland are known to remove fecal coliforms, high loads of indicator bacteria still enter Lake Victoria [36]. It is therefore possible that *Salmonella* contaminated water could have entered the lake, which is an importance source of domestic water. Environmental contamination and NTS dissemination could be mitigated by instituting best management practices at the slaughterhouses and waste treatment facilities, improving sanitation in the city, and restoring Nakivubo wetland.

Two of the three WSP performed proper waste treatment evidenced by absence of *Salmonella* in effluent. This may be attributed to proper construction, maintenance, and the existence of three and four connected settling ponds which allow for adequate retention and treatment. Conversely, detection of *Salmonella* in effluent from W1 could be attributed to malfunctioning with age, use of only two stabilization ponds, overloading, and pond coverage with weeds and algae; hence inadequate waste treatment. Properly constructed and managed WSP provide acceptable sewage treatment for middle and high income communities [16].

*Salmonella* prevalence in poultry from live bird markets (2‒11%) is comparable to that on poultry farms 11% (2‒26%) in Nigeria [8]. The poultry markets we sampled receive poultry from backyard poultry keepers and commercial farms. The movement and mixing of poultry and the use of cages possibly without proper cleaning and disinfection may enhance pathogen dissemination. Also, the practice of slaughtering and dressing poultry in households may contaminate kitchen areas and enhance NTS transmission.

We evaluated resistance to individual antimicrobials and linked resistance. In this study, LCA simplified resistance data and helped to reveal the main phenotypic structures in *Salmonella* from the different sources. The initial LCA with multiple groups showed the existence of qualitative and quantitative differences in phenotypic structure. This could be attributed to differences in serovar distribution, host or environmental factors or antimicrobial use. While the number of resistance classes in *Salmonella* from the environment, human and poultry sources were different, the “SSuT+quinolones” class was common to these sources. Also, the “quinolones” class, and similar classes (“SuSxTNa” and “SSuSxTNa”) occurred in *Salmonella* from poultry and human sources. The similarities in AMR structure suggests resistance determinants or resistant bacteria could have disseminated across different sources.

*S*. Kentucky from poultry exhibited various resistance levels ranging from single–drug resistance to deca–resistance, while *S*. Kentucky from human and environmental sources showed resistance to up to 5 antimicrobials. Also, unlike *S*. Kentucky, other serovars from poultry exhibited no or low level resistance. These findings could be explained by host or management factors associated with poultry production. Alternatively, *S*. Kentucky may be prone to acquiring resistance determinants compared to other serovars.

Poultry are recognized as the main reservoirs of multi‒drug resistant *S*. Kentucky isolated from human illnesses [37,38]. Most of the *S*. Kentucky we isolated from the various sources had similar PFGE and resistance patterns (“SSuT+ quinolones” and “quinolones”), suggesting that clonal dissemination occurred between sources. *S*. Kentucky with similar PFGE patterns but with a deca‒resistance phenotype was only found in poultry, suggesting poultry could be reservoirs of a highly resistant clone. There are ongoing concerns about the international spread of a ciprofloxacin resistant clone (MLST 198) of *S*. Kentucky in European travelers returning from certain African countries [37] The same clone of *S*. Kentucky was isolated from human patients in Canada and USA, with travel history to African or Asian countries. That clone is believed to have originated from Africa where it is reported to be widely spread [39]. *S*. Kentucky isolates from our study were also highly resistant to ciprofloxacin (86.9%), raising concerns of limited treatment options and increased disease burden should human infections occur.

Whereas the mechanisms and factors driving resistance in this study remain undetermined and need to be explored, a recent study that used whole genome sequencing and comparative genome analysis of predominant *Salmonella* serovars (Typhimurium, Enteritidis, Hadar, Heidelberg and Kentucky) in broiler chickens in Canada found chromosome encoded multidrug resistance efflux pumps regardless of AMR profile, AMR genes, and class 1 integrons; and notably, *S*. Kentucky isolates had the highest number of antimicrobial and metal resistance genes [40]. Also, class 1 integrons and multi-drug resistance genes located on chromosomes and/or plasmids have been detected in *Salmonella* from Uganda; hence clonal dissemination in the absence of antimicrobial selection pressure, and/or horizontal gene transfer is possible [41].

*S*. Stanleyville was fairly common in this study, and it is reported to be common and associated with invasive disease in Mali, West Africa [42]. We found this serovar in archived human clinical isolates, highlighting its clinical importance. *S*. Stanleyville has been reported to cause urinary tract infection in a healthy boy following enteritis [43]. The concurrent detection of the same *S*. Stanleyville PFGE patterns in WTP effluent and downstream in N2 suggests the WTP is likely a point source of environmental contamination. Finding indistinguishable PFGE pattern in archived human clinical isolates, ruminants, human wastewater and NC suggests there could have been persistent clonal dissemination among diverse sources. A recent study found *S*. Stanleyville in piglets in northern Uganda [44] but we did not find this serovar in swine.

Consistent with existing knowledge, *S*. Newport was commonly found in ruminants and swine which are regarded as reservoirs. Finding indistinguishable *S*. Newport PFGE pattern in environment, cattle and human sources suggests transmission occurred between these sources. Whereas we observed few PFGE patterns in *S*. Newport, PFGE has been shown to discriminate between *S*. Newport isolates from various sources well. The ability of PFGE to discriminate depends on serovar, number of isolates, source, and other factors [45].

Unlike studies in USA where *S*. Newport is associated with cephalosporin resistance,[46], *S*. Newport and other *Salmonella* from ruminants and swine in this study were mostly pan–susceptible. These findings suggest unlike studies where food animals are considered reservoirs of resistant NTS [47], ruminants and swine in our study may not be a significant source of resistant NTS for humans. Although information on antimicrobial use in Uganda is limited, livestock production is still predominantly traditional or less intensive and antimicrobial use in cattle and swine appears to be minimal [48].

The PFGE and AMR patterns observed in *S*. Haifa indicate transmission occurred between poultry and humans, and similar strains persisted in the environment and human population. In this study, *S*. Senftenberg recovered from human and environmental sources were mostly pan‒susceptible. However, a study from Zambia reported severe human infections with an extensively drug resistant clone of *S*. Senftenberg [49].

We did not find *S*. Typhi, and only recovered one *S*. Paratyphi B isolate. The culture media we used is reported to inhibit growth of *Salmonella* serovars that cause typhoid or paratyphoid fever [50]. A study in Uganda reported *S*. Enteritidis and *S*. Typhimurium were the main cause of bacteremia in malnourished children, while *S*. Typhi was isolated from only 5 out of 76 cases [51].

Whereas *S*. Enteritidis and *S*. Typhimurium were common in archived human isolates, we did not find *S*. Enteritidis, and only detected two *S*. Typhimurium isolates from human sources. A study that failed to recover *S*. Enteritidis and *S*. Typhimurium in surface waters despite presence of these serovars in livestock attributed it to lower survival in natural environments [52]. We had expected to find more *S*. Enteritidis and *S*. Typhimurium because these serovars cause most NTS gastroenteritis cases in humans worldwide [53] and bacteremia in Africa [14]. To assess genetic relatedness and to infer transmission in *S*. Enteritidis and *S*. Typhimurium, clustering of MLVA genotypes was performed. The clustering of *S*. Typhimurium genotypes originating from archived poultry and human clinical cases, human wastewater and the environment suggests there was persistent dissemination between diverse sources. The occurrence of a clonal complex containing archived *S*. Enteritidis isolates collected from humans in 2007 and 2010 suggests a clone persistently circulated in humans. The clustering of poultry isolates collected in 2003 and 2010, human clinical isolates collected in 2010, and poultry and environmental samples collected in 2012 suggests clonal dissemination occurred across these sources over several years.

The prevalence of *Salmonella* in pigs in our study is low compared to a study that sampled piglets and weaners from herds in northern and eastern Uganda [44]. Also, the serovars from that study and our study were different and could be attributed to age or regional differences.

Whereas the inclusion of archived isolates enabled us to link genotypes and AMR phenotypes in contemporary and archived *Salmonella* to gain insights in transmission and persistence, there were differences in sampling strategy, sources, number of isolates and representability.

We inferred NTS transmission based on AMR phenotypes and DNA fingerprinting methods (MLVA and PFGE). This is a potential limitation in that whole genome sequencing could have provided better resolution for determining phylogenetic relationships for inferring transmission. Despite this limitation, we showed that shared genotypes and AMR phenotypes were found in NTS from human, livestock and environmental sources. Our results suggest zoonotic and environmental transmissions most likely occur, and previous studies indicate waterborne [1], zoonotic [8–10] or human-to-human transmissions occur [6]. Taken together, these findings are consistent with the hypothesis that NTS is transmitted from food animals to humans via food or contact, then, it circulates within the human community, with some dissemination to the environment due to inadequate sanitation, and backflow to humans by the waterborne and environmental routes. Another hypothesis is that the predominant serovars that cause invasive disease circulate within the human community, with some dissemination to the environment and backflow to humans. Follow up epidemiological studies using whole genome sequencing approaches would provide evidence for or against these hypotheses. Also, the AMR mechanisms and factors driving AMR, particularly in poultry need to be elucidated. Information from this study could be used for the control of NTS transmission.

## Supporting Information

S1 File *Salmonella* isolates collected from human, livestock and environmental sources in Kampala, Uganda. Snum, *Salmonella* bank number; WTP, wastewater treatment plant; WSP, waste stabilization pond system; A, ampicillin; C, chloramphenicol; K, kanamycin; S, streptomycin; Su, sulfisoxazole; Sx, trimethoprim/sulfamethoxazole; T, tetracycline; Amc, amoxicillin/clavulanic acid; Na, nalidixic acid; Cip, ciprofloxacin.
(XLSX)

S2 File Archived *Salmonella* isolates from humans and livestock in Uganda. Snum, *Salmonella* bank number; A, ampicillin; C, chloramphenicol; Gen, gentamicin; K, kanamycin; An, amikacin; S, streptomycin; Su, sulfisoxazole; Sx, trimethoprim/sulfamethoxazole; T, tetracycline; Amc, amoxicillin/clavulanic acid; Na, nalidixic acid; Cip, ciprofloxacin; Xnl, ceftiofur; Fox, cefoxitin; Tax, cefotaxime. ^a^ Antimicrobial categorized as susceptible or resistant based on Clinical Laboratory Standards Institute definitions for *Enterobacteriaceae*
(XLSX)

S3 File Antimicrobial resistance test results of *Salmonella* isolates from Nakivubo Channel. The disc diffusion assay was performed using a panel of 15 antimicrobials and the zone of inhibition was recorded in mm. Snum, *Salmonella* bank number; A, ampicillin; C, chloramphenicol; Gen, gentamicin; K, kanamycin; An, amikacin; S, streptomycin; Su, sulfisoxazole; Sx, trimethoprim/sulfamethoxazole; T, tetracycline; Amc, amoxicillin/clavulanic acid; Na, nalidixic acid; Cip, ciprofloxacin; Xnl, ceftiofur; Fox, cefoxitin; Tax, cefotaxime. ^a^Antimicrobial categorized as susceptible or resistant based on Clinical Laboratory Standards Institute definitions for *Enterobacteriaceae* or *Salmonella*.
(XLSX)

S4 File Antimicrobial resistance test results for *Salmonella* isolated from human and livestock sources. The agar dilution assay was performed using 15 antimicrobials and results are recorded as 0 (susceptible) or 1 (resistant). Snum, *Salmonella* bank number; A, ampicillin; C, chloramphenicol; Gen, gentamicin; K, kanamycin; An, amikacin; S, streptomycin; Su, sulfisoxazole; Sx, trimethoprim/sulfamethoxazole; T, tetracycline; Amc, amoxicillin/clavulanic acid; Na, nalidixic acid; Cip, ciprofloxacin; Xnl, ceftiofur; Fox, cefoxitin; Tax, cefotaxime.
(XLSX)

S5 File *Salmonella* Enteritidis multiple‒locus variable number tandem repeats analysis (MLVA) data. Seven variable number tandem repeats loci (SE1, SE2, SE3, SE5, SE6, SE8 and SE9) were used to establish MLVA genotypes.
(XLSX)

S6 File *Salmonella* Typhimurium multiple‒locus variable number tandem repeats analysis (MLVA) data. Seven variable number tandem repeats loci (ST2, ST3, ST5, ST6, ST7, ST8, and STTR10pl) were used to establish MLVA genotypes.
(XLSX)

S1 Table The proportion of *Salmonella* isolates from human, livestock and environmental sources that are resistant to a panel of 15 antimicrobials. (XLSX)

S2 Table Antimicrobial resistance profiles in *Salmonella* Kentucky from human, livestock and environmental sources. A, ampicillin; C, chloramphenicol; K, kanamycin; S, streptomycin; Su, sulfisoxazole; Sx, trimethoprim/sulfamethoxazole; T, tetracycline; Amc, amoxicillin/clavulanic acid; Na, nalidixic acid; Cip, ciprofloxacin.
(XLSX)

S3 Table Antimicrobial resistance profiles observed in serovars found in poultry. A, ampicillin; C, chloramphenicol; K, kanamycin; S, streptomycin; Su, sulfisoxazole; Sx, trimethoprim/sulfamethoxazole; T, tetracycline; Amc, amoxicillin/clavulanic acid; Na, nalidixic acid; Cip, ciprofloxacin.
(XLSX)

S4 Table Criteria used for selecting the best fitting latent class analysis model. df, degrees of freedom; G^2^, the deviance statistic; the Akaike Information Criterion; BIC, the Bayesian Information Criterion.
(XLSX)
